# In Memory of Professor Hans Jeekel

**DOI:** 10.3389/jaws.2025.15514

**Published:** 2025-10-07

**Authors:** Eva B. Deerenberg, Maarten P. Simons, Johan F. Lange

**Affiliations:** ^1^ Department of Surgery, Franciscus Gasthuis en Vlietland, Rotterdam, Netherlands; ^2^ Department of Surgery, OLVG, Amsterdam, Netherlands; ^3^ Department of Surgery, Erasmus Medical Center, Rotterdam, Netherlands

**Keywords:** research, quality of life, guidelines, hernia, musicology, surgery

**FIGURE 1:**
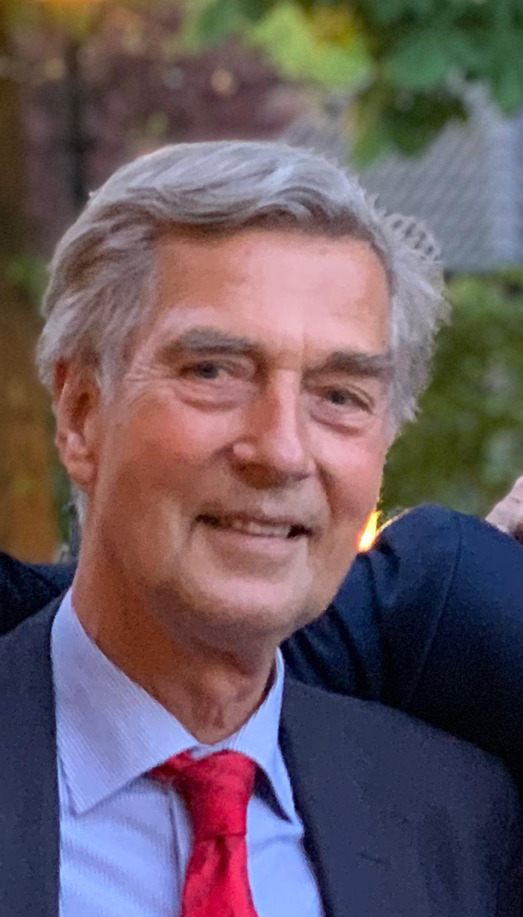
Professor Hans Jeekel (1941–2025).

On 25th July 2025 our valued, respected and loved colleague and friend professor Hans Jeekel passed away at 84 years of age (Figure 1). Hans Jeekel made a great impact on the careers and lives of many colleagues and patients with his inexhaustible and contagious efforts on improving patient outcomes by research and education. It was a privilege of knowing and working with him.

Over a remarkable career spanning more than 50 years, he has initiated and served as co-chairman of the Rotterdam Repair Group at Erasmus University with Professor Johan Lange, sr. and Professor Gert-Jan Kleinrensink, where he spearheaded and supported over 100 research projects. His efforts have resulted in more than 80 doctoral theses and over 500 publications. Many young researchers are indepted to him for a passionate and complete commitment to make them successful surgeons and scientists. At European Hernia Society congress 2025 in Paris, France Professor Hans Jeekel was awarded the prestigious EHS Lifetime Achievement Award in recognition of his extraordinary contributions to improving the welfare of patients with abdominal wall hernias.

Through a multitude of randomized controlled trials, Professor Jeekel’s work has profoundly influenced clinical guidelines on the treatment of various abdominal wall hernia types, including incisional hernias (mesh versus no mesh), umbilical hernias (mesh versus primary closure), and inguinal hernias (watchful waiting versus surgery). His research has also advanced understanding of optimal surgical techniques, biomechanical principles, and closure methods. By his efforts he greatly helped the emancipation process of abdominal wall surgery to become a full academic discipline.

He had also a profound interest in music and was a gifted pianoplayer. Although retired, in 2012 he founded a new research group: Music as Medicine at ErasmusMC. He had a vision to implement music in general healthcare. With his enthusiasm and dedication he created a very productive research team and scientifically proved that music decreases pain, anxiety, opioid use and delirium around operations, which led to an official recommendation in the Dutch guideline for peri-operative care in 2023.

Professor Jeekel’s passionate presentations at international meetings and his ability to inspire and mentor young surgeons have left an indelible mark on the field. Beyond his professional accomplishments, Hans Jeekel is celebrated for his open-mindedness, charm, graciousness, and talent as both a musician and a dancer, which many have enjoyed during hernia society gatherings around the globe.

He will be greatly missed.

